# Labyrinthectomy after Cochlear Implantation: A Case of a Novel Approach for Vertigo Control

**DOI:** 10.1155/2019/2783752

**Published:** 2019-03-31

**Authors:** I. Todt, K. Wilms, H. Sudhoff

**Affiliations:** ^1^Department of Otolaryngology, Head and Neck Surgery, Klinikum Bielefeld, Bielefeld, Germany; ^2^Department of Otolaryngology, Head and Neck Surgery, Unfallkrankenhaus Berlin, Berlin, Germany

## Abstract

Vertigo control in cases of Ménière disease and deafness can be achieved by labyrinthectomy before or as a single-stage procedure during cochlear implantation. The aim was to describe a case in which a labyrinthectomy was performed after cochlear implantation. The scar tissue was removed from the electrode cable, and the receiver was removed from the periostal pocket and placed out without electrode dislocation. Labyrinthectomy was performed after securing the electrode at the external canal. The patient disclaimed after three months no disabling vertigo. Intraoperatively, the electrode was not dislocated. A labyrinthectomy can be performed even after cochlear implantation to treat vertigo.

## 1. Introduction

Treatment of vertigo in cases of Ménière disease with functional deafness has been shown to be possible by the combination of the implant procedure with labyrinthectomy. The labyrinthectomy has been described as being performed before [1] or during the cochlear implant procedure [2, 3] to control cases of disabling vertigo and dizziness.

Less traumatic procedures are used to treat vertigo in Ménière disease cases in combination with cochlear implantation (e.g., saccus exposition, lateral semicircular canal occlusion, and triple canal occlusion) and showed good results of vertigo control [4–6].

## 2. Case

A 63-year-old man with unilateral Ménière disease (unilateral severe hearing loss, rotational vertigo attacks, dizziness episodes, caloric hypofunction, pathologic HIT, and loss of cVEMP response) and severe hearing loss and regular vestibular function of the contralateral side (caloric function, HIT, and cVEMP) was treated with an occlusion of the lateral semicircular canal, endolymphatic sac surgery, and cochlear implantation. MRI and temporal bone CT results were regular.

During a period of 9 months after the first surgery, the patient described after an initial freeness, a reoccurrence of rotational attacks, which were caused, as described by the patient, by the implanted ear and an increase of disabling dizziness. Attacks were not triggerable with a duration of up to 30 min. The attacks were independent from an electrostimulation by the cochlear implant.

**Figure 1:**
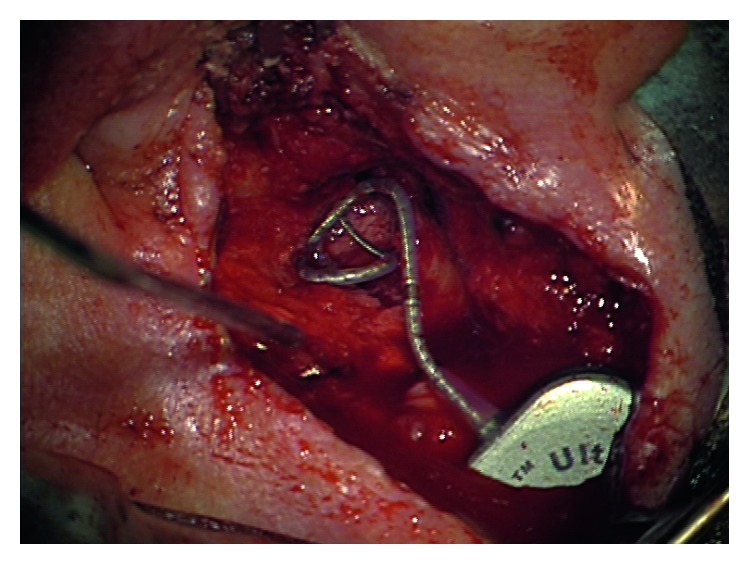
Removed electrode scar.

**Figure 2:**
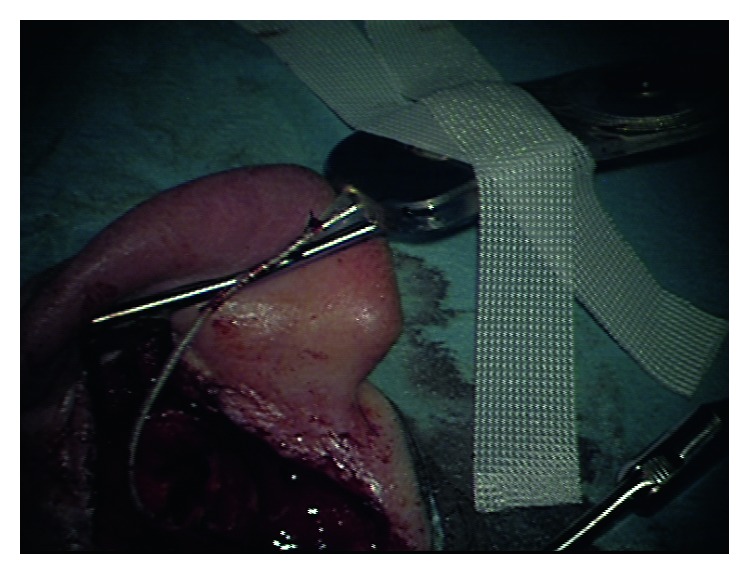
Magnet-fixed implant receiver after preparation of electrode cable.

**Figure 3:**
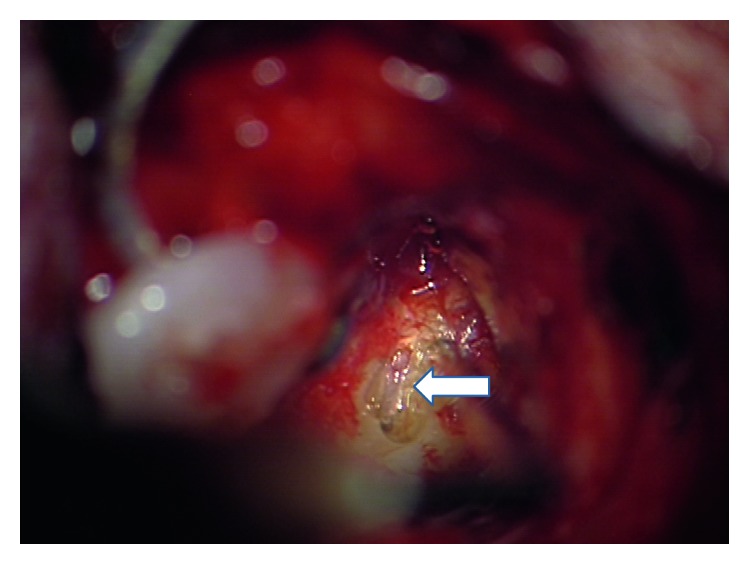
Mastoid scar removed and view on the opened lateral semicircular canal (arrow).

**Figure 4:**
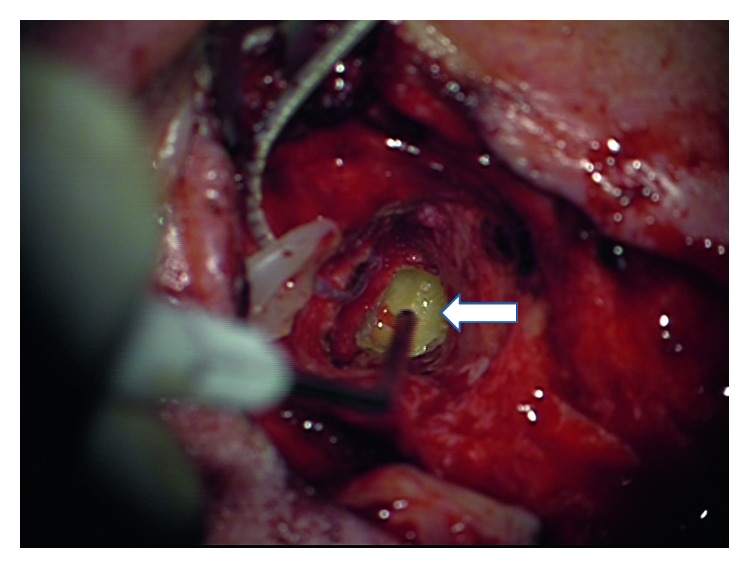
Electrode bone wax attached and view into the vestibulum (arrow).

We decided to perform a labyrinthectomy of the previously implanted side. After a very careful preparation of the electrode cable (Figure 1), the implant receiver was removed from the implant bed/periostal pocket without pulling the electrode out of the cochlea (Figure 2). The receiver was fixed by the implant magnet and Steri-Strips. The scar was removed completely from the mastoid cavity. Then, the electrode was fixed at the posterior wall of the external auditory canal with bone wax (Figure 3). After that, the labyrinthectomy was performed without dislocation of the electrode from the cochlea (Figure 4). During the labyrinthectomy, the complete occlusion of the lateral semicircular canal was confirmed. Finally, the implant receiver was placed back into the implant bed. The response thresholds of the electrically evoked compound action potential (NRI) on all implanted electrode contacts do not differ before and after surgery. Related to the regular NRI response, an X-ray was done the next day, which confirmed that the intracochlear insertion depth was the same as preoperatively. NRI threshold after the first surgery for electrodes 1, 6, 11, 16 was 153 CU, 135, 173, and N/A and after the second surgery was 123 CU, 151, 170, and N/A. These small changes indicate a minimal movement of the electrode.

Hearing results before and after the labyrinthectomy with the cochlear implant were 50% monosyllabic speech perception at 65 dB 6 weeks after the second surgery.

The study was approved by the institutional review board of Klinikum Bielefeld, Germany (IRB-klibi-HNO-2018/01). The patient provided written informed consent for the use of his clinical records.

## 3. Discussion

Treatment of vertigo after cochlear implantation is challenging because the new occurrence might subjectively give the patient the feeling of an unsuccessful overall procedure. Sometimes, the disabling vertigo cannot be balanced out against the newly gained improved hearing.

In this case, the incomplete resolution of the disabling vertigo and the unclear rotational attacks after the first procedure were successfully treated by the labyrinthectomy after the cochlear implantation. While labyrinthectomies before [1] and during cochlear implantation [2, 3] have been described previously, this is, to our knowledge, the first description of a labyrinthectomy after a cochlear implantation.

The performed surgical procedure bore the risk of a loss of the implant by cutting the electrode cable and should be carefully selected. However, carefully removing the scar from the cable was shown to be possible but time consuming (2.5 h). In future cases, a preoperative 3D radiological estimation of the cable position in the mastoid could decrease both surgical time and the cable-cutting risk. Additionally, a marker on the cable indicating the direction on the electrode cable would support the security of the surgical procedure. Since preparation of the cable during the scar removal is not performable consistently from the receiver to the posterior tympanotomy, a hardware marker on the cable would ease orientation during the preparation especially in hardly scared situations.

A second risk is that a pulling out of the electrode during the preparation can occur. We tried to limit that risk by an early attachment of bone wax on the electrode cable to fix it at the bony external auditory canal. The implanted preformed midscalar electrode might have been an additional factor that decreased the risk of a pullout in comparison to a straight electrode by an unintentional hook resistance ability inside the cochlea.

In general, this case shows that a labyrinthectomy is possible even after a previously performed cochlear implantation. Alternative procedures were not performed (transcanal labyrinthectomy and gentamicin) related to the possible risk of incomplete removal of nerval structures, iatrogenic cholesteatoma, or possible reoccurence of vertigo.
